# Comparative study on mid- and long-term clinical effects of medial pivot prosthesis and posterior-stabilized prosthesis after total knee arthroplasty

**DOI:** 10.1186/s13018-020-01951-9

**Published:** 2020-09-17

**Authors:** Weipeng Shi, Yaping Jiang, Changyao Wang, Haining Zhang, Yingzhen Wang, Tao Li

**Affiliations:** 1 Department of Joint Surgery, The Affiliated Hospital of Qingdao University, No. 59, Haier Road, Qingdao, 266000 China; 2 Medical Department of Qingdao University, Qingdao, 266071 Shandong China; 3 Department of Oral Implantology, The Affiliated Hospital of Qingdao University, Qingdao, 266003 China

**Keywords:** Medial pivot prosthesis, Posterior-stabilized prosthesis, Total knee arthroplasty, Mid- and long-term, Clinical effect

## Abstract

**Objective:**

The purpose of this study was to explore the mid-and long-term clinical effects of Chinese patients with medial pivot (MP) prosthesis and posterior-stabilized (PS) prosthesis after total knee arthroplasty (TKA), to provide a reference for the recommendation of clinical prostheses.

**Methods:**

A retrospective analysis of 802 patients who received TKA was performed from June 2010 to December 2013. A total of 432 patients received a MP prosthesis (MP group) and 375 patients received a PS prosthesis (PS group). Postoperative range of motion (ROM), clinical scores including the knee scoring system (KSS), the Western Ontario and McMaster Universities osteoarthritis index (WOMAC), the forgotten joint score (FJS), and postoperative complications were compared between the two groups.

**Results:**

A total of 527 patients were followed up, including 290 in the MP group and 237 in the PS group. Both groups achieved satisfactory results in terms of KSS score, WOMAC score, and postoperative ROM, which were significantly improved compared with those before surgery, but the difference between the groups was not statistically significant (*P* > 0.05). The FJS scores of the MP group and the PS group were satisfactory and no significant difference was observed (*P* = 0.426). Postoperative complications occurred in 5 and 11 patients in the MP group and PS group, respectively.

**Conclusion:**

The clinical results of TKA with MP or PS in Chinese patients at mid- and long-term are encouraging, and no significant differences were observed between the two types of prostheses. Studies have also shown that both prostheses are safe for Chinese patients.

## Introduction

With the development of the aging population, knee joint degenerative disease has become a global common condition of the elderly. At present, total knee arthroplasty (TKA) is the first choice for the treatment of end-stage osteoarthrosis [1, 2]. The first-generation knee prosthesis was designed and developed by Gonston et al. in 1969 [3]. After increased development and improvement, present-day knee joint prostheses can better restore the natural kinematics of the knee joint and have improved the survival rates.

Posterior-stabilized (PS) prosthesis is a classic clinical prosthesis, which has the advantages of a clear incision exposure, a simple soft-tissue balance, and a greater range of motion (ROM) of the knee joint [4]. It relies on the femoral cam to improve the rollback, improves the stability of knee joint movement and motion translation, and prevents posterior subluxation [5]. However, the structure of the cam post may impact the central post, cause patella slip syndrome, and the extent of the osteotomy is large, which introduces difficulties for subsequent revision surgery [6].

During knee flexion, the contact points of the medial tibiofemoral articular surface are used as the axis, and the tibia is rotated in relation to the femur [7–9]. According to the characteristics of knee kinematics, the medial pivot (MP) prosthesis, which adopts the concept of the “ball-and-socket” design, has been developed. The medial part of the high molecular polyethylene gasket has the shape of a “ball-and-socket” unit, which limits anterior and posterior movement of the medial condyle of the femur, while the lateral condyle can achieve normal back-rolling movement during knee flexion [4, 10–13]. The MP prosthesis with a single radius of curvature has the advantages of maximizing the ROM of the joint and the contact area between the polyethylene gasket and the femoral prosthesis. This will reduce the wear of the gasket, thereby increasing joint stability and improving the patella trajectory through the “ball-and-socket” model [14–16].

However, due to differences in anatomical structure between the Asian and European population, postoperative knee pain, limited mobility, and joint instability often occur [17–19], and there are very little experimental data obtained from Chinese people. The purpose of this study was to compare mid- and long-term clinical effects of TKA with MP and PS prostheses to provide a reference for the clinical selection of prostheses in Chinese patients.

## Materials and methods

From June 2010 to December 2013, a retrospective study of patients undergoing TKA at the Department of Joint Surgery, the Affiliated Hospital of Qingdao University (Qingdao, China) was conducted. All included patients were diagnosed with knee osteoarthropathy. Patients were excluded if they had a BMI 35 kg/m^2^, suffered from cardio-cerebral vascular, neurological, and mental related system diseases, or had previous fractures around the knee joint. Patients who received other surgical treatments, such as high tibial osteotomy, patients who intended to undergo knee revision surgery, or patients whose daily lives were severely affected due to reasons other than knee surgery, were not included in this study.

In this study, MP (Advance Medial-Pivot Knee System, Wright Medical Group) or PS prosthesis (NexGen LPS-Flex, Zimmer, Warsaw, IN) were adopted. Patients were placed into the supine position, with general anesthesia and a nerve block being used before surgery, and electric pneumatic hemostat as well as 300 mmHg pressure was used at the start of surgery. An anterior median and longitudinal incision of the knee joint was created, and the paracondylar approach was used to remove the synovium, part of the fat pad, the meniscus, the anterior cruciate ligament, and osteophytes. The femoral side was positioned intramedullary, the distal femur at valgus 5° (PS prosthesis 6°), the tibial side was placed extramedullary, and the posterior incline was 3° (PS prosthesis 7°). The patella was trimmed, and the local anesthetic was injected into the surrounding tissue and posterior articular capsule before the prosthesis was placed. Tranexamic acid was injected into the articular cavity before suturing the subcutaneous tissue and a drainage tube was inserted.

By searching the electronic medical record system of the Affiliated Hospital of Qingdao University (Qingdao, China), data on the knee movements of patients before surgery were obtained, and all data obtained after surgery were measured by specialists in joint surgery. The patient was placed in a supine position, and the knee joint was straightened and flexed as much as the patient tolerated. Extension and flexion angles were recorded. For each patient, the measurement was performed in triplicate, and the final angle was averaged. The KSS score scale is divided into two parts: the clinical and the functional score, with the total score of both parts being 100 points. Scores greater than 85 points are considered excellent, 70-84 points are good, 60-69 points are acceptable, and scores less than 60 points are considered poor. Responses on the WOMAC scoring scale can be rated as “no difficulty,” “slight difficulty,” “medium difficulty,” “very difficult,” or “extremely difficult,” with a score of 0–4 points, and the total score is the sum of the individual scores. The FJS score is a scale for evaluating a patients’ post-operative satisfaction. It includes 12 questions with five possible answers for each question. A high total score indicates a high degree of forgetting and a low score indicates a low degree of forgetting. Complications mainly include pain in the anterior knee area, loss of sensation around the knee joint incision, knee snapping and poor healing of the incision, fracture around the prosthesis, loosening of the prosthesis, biomechanical instability of the knee joint, infection around the prosthesis, and other related systemic complications.

The SPSS software (version 26.0) was used to analyze the collected data, which were tested by Kolmogorov-Smirnov test for normal distribution, *t* test, non-uniform normal distribution, and non-parametric test. The chi-square test was used for comparison between groups, and the significance level was set at 0.05.

## Results

**Fig. 1:**
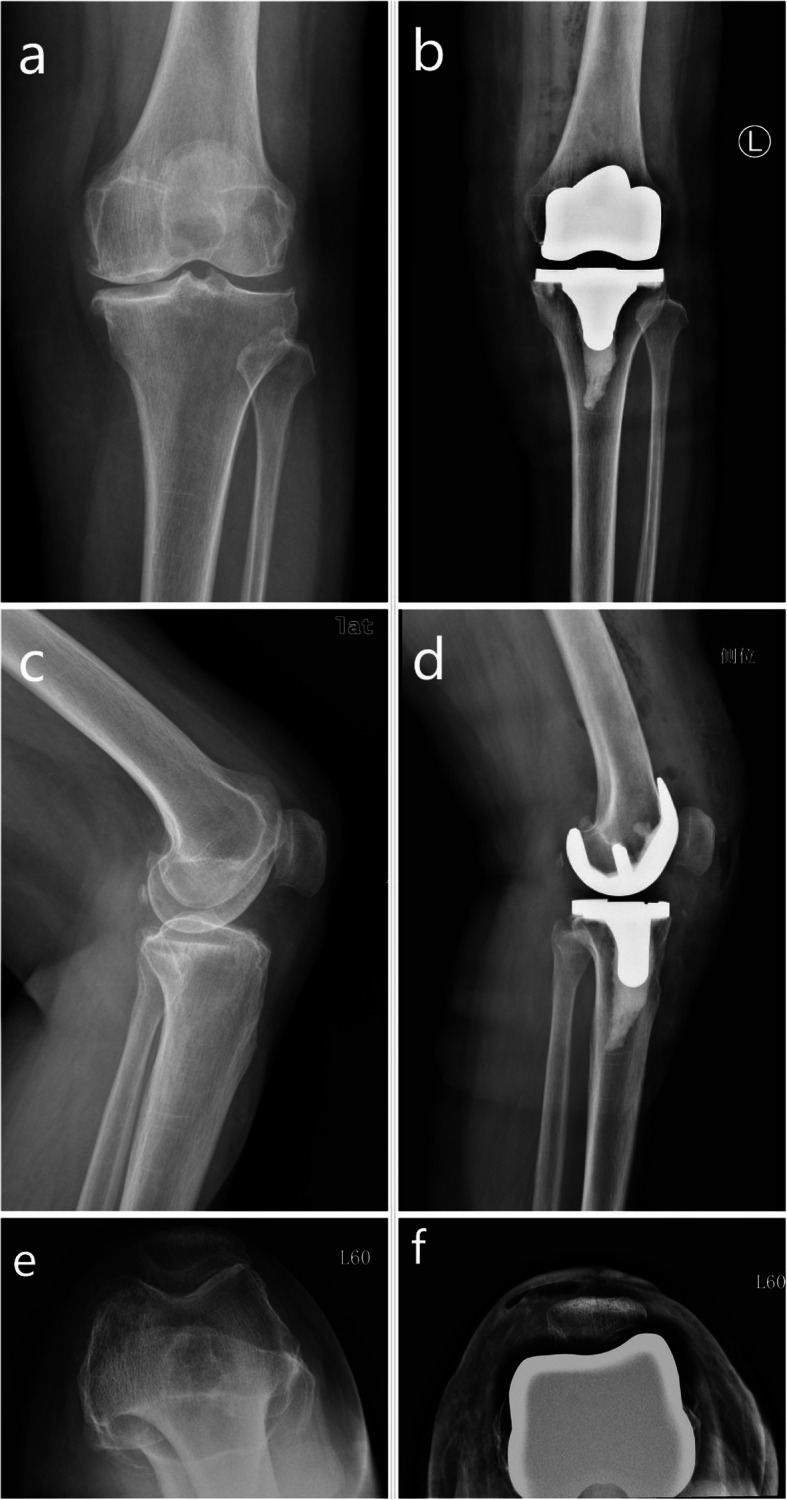
Typical case of medial pivot prosthesis. **a** Anteroposterior position before surgery in patients with medial pivot (MP) prosthesis. **b** Anteroposterior position after surgery in patients with MP prosthesis. **c** Lateral position of the knee joint before surgery in patients with MP prosthesis. **d** Lateral position of the knee joint after surgery in patients with MP prosthesis. **e** Patella axial radiograph (60°) before surgery in patients with MP prosthesis. **f** Patella axial radiograph (60°) after surgery in patients with MP prosthesis

**Fig. 2:**
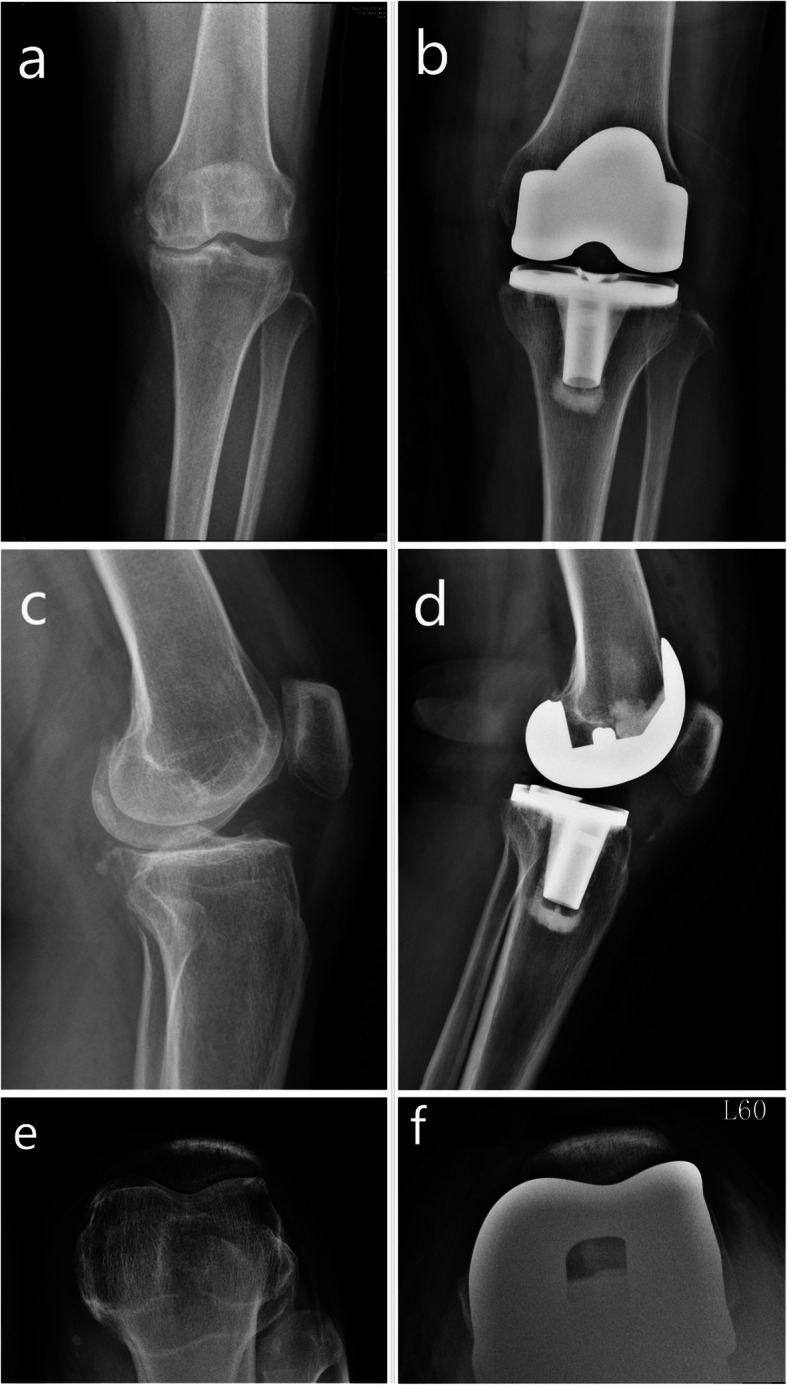
Typical case of posterior-stabilized prosthesis. **a** Anteroposterior position before surgery in patients with posterior-stabilized (PS) prosthesis. **b** Anteroposterior position after surgery in patients with PS prosthesis. **c** Lateral position of the knee joint before surgery in patients with PS prosthesis. **d** Lateral position of the knee joint after surgery in patients with PS prosthesis. **e** Patella axial radiograph (60°) before surgery in patients with PS prosthesis. **f** Patella axial radiograph (60°) after surgery in patients with PS prosthesis

This study included 802 patients who met the inclusion criteria, and a total of 527 patients for whom follow-up data were available. In the MP group, 290 patients were followed up. The average age of the patients was 74.58 (± 6.97) years, the average body mass index (BMI) was 27.89 (± 3.65) kg/m^2^, and the average follow-up time was 81.04 (± 7.66) months. In the PS group, 237 patients were followed up. The average age of the patients was 75.84 (± 5.70) years, the body mass index (BMI) was 27.43 (± 3.51) kg/m^2^, and the average follow-up time was 80.78 (± 7.85) months. Both groups were balanced in their baseline characteristics (Table 1). Typical cases of MP prosthesis and PS prosthesis are presented in Figs. 1 and 2.

**Table 1:** Preoperative characteristics of patients with follow-up data

	MP^b^ group (*n* = 290)	PS^c^ group (*n* = 237)	Statistics	*P* values
Gender (female)	228 (78.6%)	169 (71.3%)	3.753	0.053
Age (year)	74.5 ± 6.97	75.4 ± 5.70	31,359.500	0.084
Body mass index (kg/m^2^)	27.89 ± 3.65	27.43 ± 3.51	32585.000	0.306
Side (left)	140 (48.3%)	110 (46.4%)	0.181	0.053
Length of follow-up (month)	81.04 ± 7.66	80.78 ± 7.85	33,509.000	0.622
KSS^a^ clinical score	40.76 ± 10.00	41.35 ± 7.50	33,675.500	0.692
KSS function score	41.00 ± 11.44	41.18 ± 11.20	34,013.000	0.437
WOMAC^d^ total score	80.72 ± 6.35	80.04 ± 7.09	32,166.000	0.206
WOMAC pain score	15.00 ± 3.23	14.98 ± 4.08	34,117.500	0.885
WOMAC rigidity score	5.93 ± 1.34	5.92 ± 1.65	33,944.000	0.800
WOMAC activity score	59.19 ± 6.35	59.14 ± 5.27	32,054.500	0.183

Comparison of the main indicators of the two groups

^a^*KSS* knee scoring system

^b^*MP* medial pivot prosthesis

^c^*PS* posterior-stabilized prosthesis

^d^*WOMAC* the Western Ontario and McMaster Universities osteoarthritis index

### Rom

**Fig. 3:**
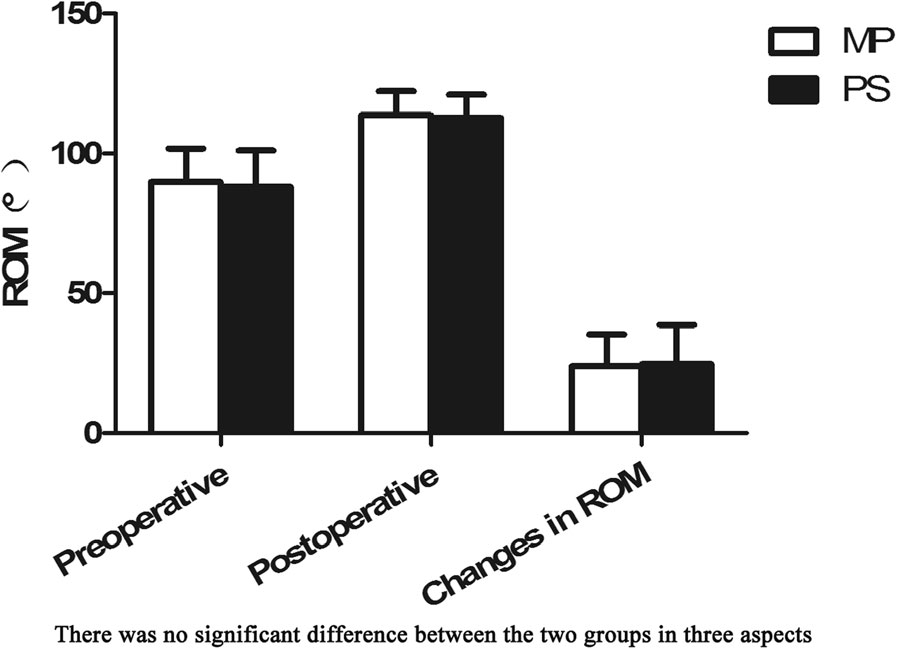
Preoperative/postoperative changes in range of motion. There was no significant difference between the two groups in preoperative ROM, postoperative ROM, and changes in ROM. *P < 0.05

The average preoperative ROM was 89.79 (± 12.00) in the MP group and 88.04 (± 13.02) in the PS group. The postoperative ROM of the MP and PS groups were 113.72 (± 8.43) and 112.72 (± 8.18), respectively, with no significant difference between the two groups (*P* = 0.253). The ROM of the MP group increased by 23.93 (± 11.39) and the ROM of the PS group increased by 24.68 (± 14.05) compared to before the operation. No significant differences were observed between the two groups (*P* = 0.978) (Fig. 3).

### KSS

**Fig. 4:**
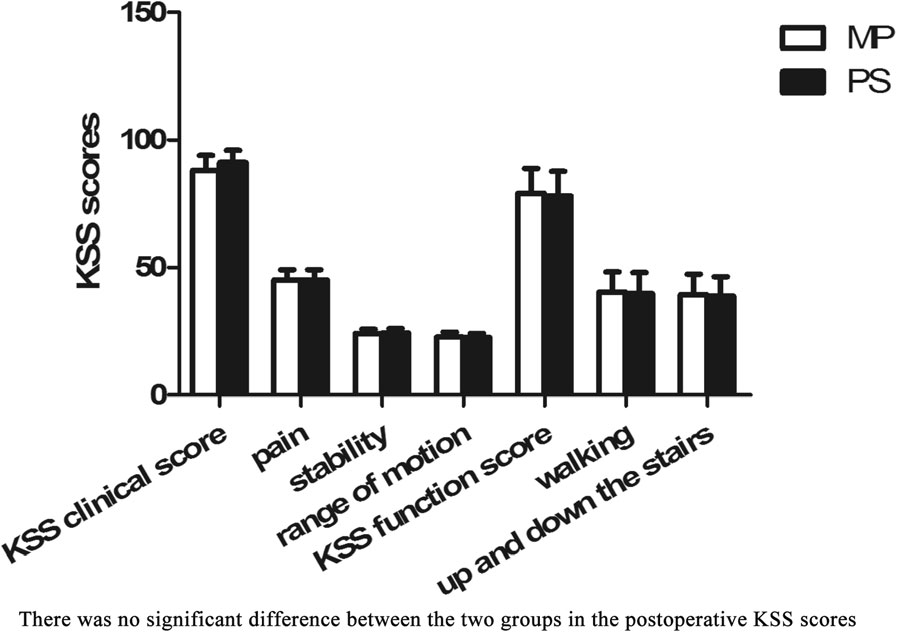
Postoperative knee scoring system (KSS) score. There was no significant difference between the two groups in the postoperative clinical and functional scores of KSS. *P < 0.05

The clinical and functional scores of the KSS before surgery in the two groups were balanced (*P* > 0.05) (Table 1). Scores related to pain, stability, walking conditions, and up and down the stairs of the two groups of patients were significantly improved after surgery, but no significant differences were observed between the clinical and functional scores of KSS (Fig. 4).

### WOMAC

**Fig. 5:**
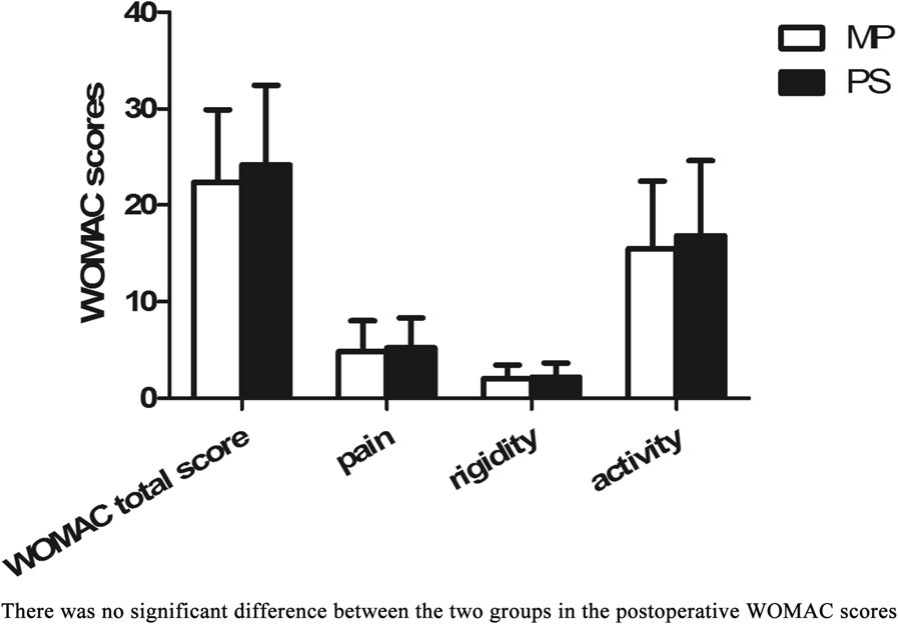
Postoperative Western Ontario and McMaster Universities osteoarthritis index (WOMAC) score. The difference between the postoperative WOMAC scores of the two groups was not statistically significant. *P < 0.05

Patients in the MP and PS groups had significantly lower postoperative WOMAC scores in terms of pain, stiffness/rigidity, and activity/daily life than before the surgery. Among them, the activity score improved the most, however, the differences between the WOMAC scores of the two groups was not statistically significant (Fig. 5).

### FJS

The overall FJS score between the two groups was satisfactory, with an average of 68.89 (± 25.04) in the MP group and 65.29 (± 24.93) in the PS group. The degree of amnesia of the knee prosthesis in the MP group was slightly higher compared to that in the PS group, but no significant differences were observed in the FJS scores between the two groups (*P* = 0.426).

### Complications

In the MP group, five patients experienced complications from the surgery. Two patients had a surgery-related infection, but this subsided after the second-stage revision. One patient experienced joint pain, which was treated by polyethylene gasket removal. One patient experienced numbness around the incision, and neurotrophic drugs was adopted. The patient still feels insensitive in certain areas around the knee. During the recovery phase, in one patient, the incision was not healed 2 weeks after surgery. Finally, it was healed through multiple surgical dressings. In the PS group, a total of 11 patients showed complications, one person had an infection after surgery but recovered after the two-stage revision, and three people presented with pain in the anterior knee area during postoperative activities, which gradually disappeared after oral administration of non-steroidal anti-inflammatory drugs. The knee joint of five patients clicked slightly, and two patients presented with numbness around the incision, which did not affect daily life; hence, no targeted treatment was given. No systemic complications, such as deep vein thrombosis or pneumonia in the lower extremities were observed.

## Discussion

This study aimed to explore the clinical results of Chinese patients who underwent implantation of MP and PS prostheses for TKA at mid- to long-term follow-up. We found that MP and PS prostheses have similar clinical effects mid- to long-term, both for knee function and ROM. This overall effect was consistent with the findings presented in previous studies, which showed that MP prostheses can achieve satisfactory results in clinical and imaging studies at mid- to long-term follow-up [4, 8, 20–22]. Chinzei et al. [20] found that the success rate of the MP prosthesis was 98.3% during an 8-year follow-up of 76 patients (85 knees), however, only one case of infectious loosening occurred. The survival rate of the prosthesis in the study of Dehl et al. [21] reached 95.9%, and no aseptic loosening was observed. In the present study, two cases in the MP group presented with an infection around the prosthesis after surgery, which was successfully treated after the second-stage revision, and one case was treated after removal of the polyethylene gasket due to joint pain. There was one case of joint infection in PS group, which was adequately treated after the second-stage revision. The 6.5-year survival rate of the PS prosthesis was about 99.6%. In the PS group, there was one infection, which was recovered after a second-stage revision. The infection rates of the MP and PS groups were 0.7% and 0.4%, respectively, which were similar to the results presented in the previous studies [23]. In this study, the 6.5-year survival rate of the MP prosthesis was slightly lower than that of the PS group. It cannot be ruled out that there may be data deviations caused by various factors, such as revisions or surgical techniques in missing patients. Compared with the PS prosthesis, the MP prosthesis produced smaller, rounder, and fewer particles, which may have a smaller effect on osteolysis and aseptic loosening [24], making the MP prosthesis more ideal in terms of the survival rate.

In this study, no significant differences in both the postoperative ROM and ROM increase were observed between the two groups, which was similar to the findings presented in the previous studies [25, 26]. Although the MP prosthesis may have advantages over the PS prosthesis in kinematics and contact area, no significant differences were observed between the two groups in terms of postoperative flexion. David et al. [4] found that the MP prosthesis had a better postoperative flexion than the PS prosthesis (MP group 120.3° vs PS group 112.8°). In only a few studies, it was shown that the postoperative flexion of the MP prosthesis can reach more than 120°, for example, in a study by Bae et al. [26], 150 patients with an MP prosthesis were followed up for 5 years, and it was found that the average postoperative flexion can reach 124°, which may be related to the preoperative flexion, reaching more than 120°. However, the follow-up of 92 patients by Kim et al. [27] for 2.6 years showed that the early clinical effect of MP prosthesis was worse with a smaller ROM, poor patient satisfaction, and higher incidence of complications.

In this study, both MP and PS groups showed a significant improvement in the KSS scores compared to the scores before surgery, which was similar to the results previously reported [20, 26, 28]. The rates of the postoperative KSS clinical score in both groups reached 100%, and the rates of KSS functional score were 86.8% and 86.9%, in MP and PS groups, respectively, which may be related to a more subjective index items in the functional score. In the WOMAC score, the two groups of patients also improved significantly, but there was no significant difference between groups. At present, there are a few studies on the use of the FJS, which has the advantages of a tool with high structural validity and high reliability for repeated testing, with the upper limit effect of the FJS being lower compared to WOMAC. In previous studies, patients in the MP group had a higher final FJS score than the PS group [4, 29], because a high degree of stability is required when the knee joint is straightened from the flexion state. In contrast, the cam mechanism of the PS prosthesis will produce higher contact stress, which leads to knee instability, thereby affecting the ability of the knee joint to change from high flexion to straight extension [30]. Therefore, the FJS score in the PS group was lower than that in the MP group.

Current research suggests that skin numbness and paresthesia after TKA are mainly caused by cutaneous nerve injury close to the anterior knee incision [31]. The peripheral nerve distribution of the knee joint is divided into shallow and deep layers. The shallow layer comprises the cutaneous nerve, and the deep layer is composed of the joint capsule, the surrounding ligaments, and the arterial branches that are connected to the joint. Clinically, the classic approach of anterior midline incision of the knee joint is mostly used, therefore, it is inevitable that the cutaneous nerve in front of the knee joint is cut, resulting in numbness of the skin around the cut after the artificial TKA. In the present study, one patient in the MP group and two in the PS group experienced numbness around the incision, because the surgery was carried out by creating a mid-knee incision, resulting in a lack of sensation due to damaged cutaneous nerves. The affected patients received neurotrophic drugs for conservative treatment, but in most cases, partial sensory deficits remained.

Pain in the anterior knee is a major factor affecting the quality of life of patients after TKA. It can be caused by dysfunction of the patellofemoral joint in the anterior knee region, abnormalities in the patellar trajectory, and high contact stress of the patellofemoral joint after surgery [32]. In previous studies, it was shown that pain in the anterior knee area was closely related to the type of prosthesis [33, 34]. The PS prosthesis requires an intercondylar box to accommodate the column, and when the knee joint changes from flexion to extension, the patella will touch the intercondylar box. The long-term consequences of this constellation are hyperplasia of the fibrous tissue nodules, which get in contact with the intercondylar box of the PS prosthesis, and cause pain in the anterior part of the knee during the extension to 30–40° [35, 36]. In the present study, three patients in the PS group developed pain in the anterior knee area. After continued oral administration of nonsteroidal anti-inflammatory analgesics for 2 months, the symptoms gradually disappeared, without adversely affecting postoperative functional recovery.

Clinically, the phenomenon of joint popping or a clicking sound after knee arthroplasty is quite common. The main reason is that small nodules of the fibrous synovial hyperplasia at the superior junction of the quadriceps tendon and patella are stuck in the intercondylar fossa during movement of the knee joint. The box restricts the upward movement of the patella. When the small nodule pops out of the intercondylar box, the patella suddenly moves upwards and produces a snapping sound [37, 38]. Hozack coined this phenomenon the patellar clunk syndrome, which may be related to an abnormal patella trajectory and the type of prosthesis [38–42]. The average time for the clicking sound to occur for the first time is 5 to 11 months [43]; however, it has also been reported that it can occur up to 6 years after surgery [44] and generally requires surgery to be removed. Anderson et al. [17] pointed out that because of the improvement of the MP prosthesis biomechanically, it does not require an intercondylar box to accommodate the post, which can extend the pulley groove downward so that the patella does not leave the femoral pulley during extension and flexion, thereby reducing the occurrence of patellar and postoperative patellofemoral joint complications from 25 to 0% in the PS group. In the MP group, no postoperative clicking sound was observed, while five patients with slight clicking presented in the PS group, which occurred mainly during the process of knee joint flexion to extension. We suspect that it may be caused by hyperplastic nodules that are stuck in the intercondylar box. Because these patients felt it did not affect their daily life, they were not given targeted diagnosis and treatment.

In summary, despite the high loss to follow-up rate in this study, no significant differences were observed between the two types of prostheses in terms of postoperative clinical effects, infection rates, and prosthesis survival rate in Chinese patients. The overall improvement is encouraging in at mid- and long-term follow-up after surgery, and patient satisfaction is high. The incidence of postoperative complications in general is lower than the overall level, which shows that the two types of prostheses are safe and reliable.

## Conclusion

The clinical results of knee arthroplasty with MP or PS in Chinese patients at mid- and long-term follow-up are encouraging, with no significant differences observed between the two types of prostheses. Studies have also shown that both prostheses are safe for Chinese patients.

## Data Availability

All data are contained in the text and charts of published articles.
